# Comparison of Two High‐Power Ablation Strategies for Typical Atrial Flutter: Acute and Long‐Term Outcome

**DOI:** 10.1111/anec.70089

**Published:** 2025-05-29

**Authors:** Wael Zaher, Lorenzo Marcon, Klaus‐Richard Ebinger, Antonio Sorgente

**Affiliations:** ^1^ Department of Cardiology Centre Hospitalier EpiCURA Hornu Belgium

**Keywords:** cavo‐tricuspid isthmus ablation, high‐power, typical flutter

## Abstract

**Background:**

Ablation of the cavo‐tricuspid isthmus (CTI) is the standard treatment for typical atrial flutter. High‐power strategies have been described to improve lesion efficacy and durability.

**Objective:**

To compare the acute success, safety, and long‐term outcomes of two strategies of high‐power CTI ablation using 8‐mm gold‐tip nonirrigated and 4‐mm irrigated‐tip catheters.

**Methods:**

This single‐center prospective cohort study included 253 patients who underwent CTI ablation. Patients were treated with either an 8‐mm gold‐tip nonirrigated catheter (60 W, ≥ 30 s) or a 4‐mm irrigated catheter (45 W, ≥ 30 s). Procedural outcomes, safety, and long‐term follow‐up data were assessed.

**Results:**

Using a propensity score matching, 180 patients were yielded with a 1:1 ratio. Acute bidirectional CTI block was achieved in 97.8% of the 4‐mm group and 97.8% of the 8‐mm group (*p* = 1.000). No major complications were reported. During a median follow‐up of 27.7 ± 20.1 months, freedom from atrial arrhythmia was 93.3% in both groups (log rank *p* value 0.935). No significant differences were observed in atrial fibrillation incidence, pacemaker implantation, or cardiovascular mortality between the groups.

**Conclusion:**

High‐power CTI ablation with both 8‐mm gold‐tip nonirrigated and 4‐mm irrigated catheters is highly effective and safe, providing durable outcomes over long‐term follow‐up.

## Graphical abstract

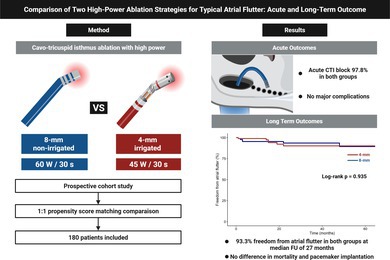

## 1 Introduction

Typical atrial flutter is a macro‐reentrant arrhythmia characterized by a circuit around the tricuspid annulus, involving the cavo‐tricuspid isthmus (CTI). Radiofrequency catheter ablation targeting the CTI aims to achieve a durable bidirectional conduction block to prevent recurrent atrial flutter episodes (Brugada et al. 2020). Contemporary approaches utilize irrigated‐tip and nonirrigated large‐tip catheters, both designed to create more effective lesions than the conventional 4‐mm tip catheters (Jais et al. 2000; Tsai et al. 1999). Common power settings include 50 W for 8‐mm nonirrigated catheters and 30 W for irrigated‐tip catheters. Recently, alternative strategies with irrigated‐tip catheters have emerged, employing high power (e.g., 50 W for short durations of 9–20 s) (Golian et al. 2020; Kwon et al. 2020; Tscholl et al. 2020) or very high power for very short durations (e.g., 90 W for 4 s) (Schillaci et al. 2022). Our study evaluates the acute safety and efficacy, as well as long‐term outcomes, of two high‐power strategies using both nonirrigated and irrigated‐tip catheters.

## 2 Methods

### 2.1 Study Design

This was a nonrandomized prospective cohort study conducted at a single‐center hospital (CH EpiCURA, Hornu, Belgium). The study was approved by the local Ethics Committee (reference number P2022/042).

### 2.2 Study Population

Patients who underwent CTI radiofrequency ablation for CTI‐dependent atrial flutter between January 2019 and December 2023 were included. Ablation was performed using high‐power, standard‐duration settings with either an 8‐mm gold‐tip nonirrigated catheter or a 4‐mm irrigated‐tip catheter, with the choice of catheter based on operator preference. Patients were categorized into two groups: the 8‐mm nonirrigated catheter group (8‐NIC) or the 4‐mm irrigated catheter group (4‐IC). Crossover cases were excluded. Ablation was restricted to the CTI without additional ablations, such as pulmonary vein isolation.

### 2.3 Ablation Procedure

Procedures were performed under general anesthesia. Continuous monitoring of surface electrocardiograms and intracardiac bipolar electrograms was conducted using a recording system. Fluoroscopy or a 3D electroanatomical system (Ensite Velocity NavX, Abbott St. Jude Medical) was used when available. Ultrasound‐guided puncture facilitated three femoral venous access sites. A steerable decapolar catheter was positioned in the coronary sinus, and a deflectable duodecapolar catheter was placed in the right atrium. Ablation was performed using either a 4‐mm irrigated catheter (Therapy Cool Flex, Abbott) or an 8‐mm gold‐tip nonirrigated catheter (AlCath LT G FullCircle 8 mm, Biotronik). Two operators performed all ablations using point‐by‐point radiofrequency delivery. Energy was applied at 45 W for a minimum of 30 s with the 4‐mm irrigated catheter or 60 W for a minimum of 30 s with the 8‐mm nonirrigated catheter, targeting a 10–15 Ω impedance drop. Lesions were initiated at the tricuspid valve and extended toward the inferior vena cava. For patients in sinus rhythm, ablation was performed during atrial pacing at 600 ms from the proximal pole of the coronary sinus. Procedure success was defined as achieving bidirectional block along the CTI, confirmed by pacing maneuvers. Clockwise conduction block was verified by reversal of activation on the duodecapolar catheter during coronary sinus pacing. Counterclockwise block was confirmed by sequential pacing on the duodecapolar catheter and time delay measurements on the coronary sinus.

### 2.4 Follow‐up

All patients underwent transthoracic echocardiography the day after the procedure. Follow‐up visits occurred at 1, 6, and 12 months. A 24‐h Holter ECG was performed at the 1‐month follow‐up. Beyond 12 months, follow‐up was conducted by the referring cardiologist as per their discretion.

### 2.5 Outcomes

The primary efficacy outcome was defined as the achievement of bidirectional CTI block confirmed by pacing maneuvers. The safety outcome was defined as the occurrence of complications within 24 h, including hemodynamic instability, stroke, vascular access complications, or pericardial effusion. Long‐term efficacy was assessed as freedom from recurrent atrial flutter during follow‐up.

### 2.6 Statistical Analysis

Baseline characteristics are reported as mean ± standard deviation for continuous variables and as observed numbers and relative percentages for categorical variables. In the unmatched cohort, between‐group comparisons were performed with Student's *t*‐test or χ^2^ test as appropriate. Propensity scores for type of catheter were estimated by logistic regression including all pretreatment covariates plausibly related to catheter selection and outcomes (age, sex, hypertension, diabetes, smoking status, prior ablation, left‐ventricular ejection fraction, antiarrhythmic and anticoagulant therapies, etc.). Patients were matched 1:1 without replacement on the logit of the propensity score using nearest‐neighbor matching within a caliper of 0.2 standard deviations of the logit‐PS. Covariate balance before and after matching was assessed by standardized mean differences, with values < 0.10 considered acceptable.

In the matched cohort, paired *t*‐tests compared continuous covariates and outcomes; McNemar's test compared binary endpoints (acute success, complications, recurrence, pacemaker implantation, etc.). Time‐to‐event analyses for recurrence and survival were performed using Kaplan–Meier estimates and compared by the log‐rank test. All significance tests were two‐tailed, with a *p* value < 0.05 considered statistically significant. Analyses were performed with R (Version 4.3.3).

## 3 Results

### 3.1 Baseline Characteristics

A total of 253 patients underwent CTI ablation. Baseline characteristics of the unmatched population are reported in Table 1. Propensity score matching based on 32 characteristics yielded 90 patients in each group. Characteristics are reported in Table 2. A well‐balanced covariate distribution among groups was observed after propensity matching. Mean age of patients was 69.4 ± 9.4 years, and 25.0% were female. Among the cohort, 32.8% had a history of heart failure with reduced ejection fraction, and 40.6% had a history of atrial fibrillation.

**TABLE 1:** Clinical characteristics.

Characteristics	All patients (*N* = 253)	4‐IC (*N* = 128)	8‐NIC (*N* = 125)	*p*
**Age**, year—mean ± SD	69.1 ± 9.9	67.9 ± 10.4	70.2 ± 9.3	0.066
**Sex**—no. (%)				
**Female**	64 (25.3)	26 (20.3)	38 (30.4)	0.089
**Cardiovascular risk factor**—no. (%)				
Arterial hypertension	179 (70.8)	88 (68.8)	91 (72.8)	0.569
Hypercholesterolemia	157 (62.1)	77 (60.2)	80 (64.0)	0.617
Diabetes mellitus	87 (34.4)	41 (32.0)	46 (36.8)	0.505
Tobacco	109 (43.1)	55 (43.0)	54 (43.2)	1.000
**Heart failure**—no. (%)				
Reduced or mid‐range ejection fraction	72 (28.4)	36 (28.1)	36 (28.8)	1.000
Preserved ejection fraction	43 (16.7)	19 (14.8)	24 (19.2)	0.450
**Coronary artery disease**	79 (31.2)	42 (32.8)	37 (29.6)	0.678
**AF history**—no. (%)	99 (39.1)	45 (35.2)	54 (43.2)	0.237
**Pacemaker**—no. (%)	36 (14.2)	16 (12.5)	20 (16.0)	0.537
**Redo CTI ablation**—no. (%)	17 (6.7)	10 (7.8)	7 (5.6)	0.651
**Medical history**—no. (%)				
Sleep apnea syndrome	39 (15.4)	22 (17.2)	17 (13.6)	0.538
Chronic obstructive pulmonary disease	61 (24.1)	29 (22.7)	32 (25.6)	0.689
Hypothyroidism–hyperthyroidism	33 (13.0)	14 (10.9)	19 (15.2)	0.412
Previous stroke history	23 (9.1)	8 (6.2)	15 (12.0)	0.170
Chronic kidney disease	68 (26.9)	32 (25)	36 (28.2)	0.589
Neoplasia history	35 (13.8)	18 (14.1)	17 (13.6)	1.000
**Echocardiography**				
Left ventricular ejection fraction, %—mean ± SD	53.29 ± 11.4	52.9 ± 12.2	53.7 ± 10.7	0.552
**Medical treatment**—no. (%)				
Antiarrhythmic class				
Class I	31 (12.3)	13 (10.2)	18 (14.5)	0.402
Class II	166 (65.9)	83 (65.3)	83 (66.9)	0.898
Class III—Sotalol	8 (3.2)	5 (3.9)	3 (2.4)	0.745
Class III—Amiodarone	59 (23.5)	29 (22.8)	30 (24.2)	0.917
Class IV	14 (5.6)	9 (7.1)	5 (4.0)	0.436
Antiplatelet	40 (15.9)	17 (13.3)	23 (18.5)	0.345
Anticoagulation				
Apixaban	40 (15.9)	20 (15.9)	20 (16.3)	1.000
Dabigatran	29 (11.5)	13 (10.3)	16 (13.0)	0.644
Edoxaban	85 (33.9)	49 (38.9)	36 (29.3)	0.143
Rivaroxaban	67 (26.7)	34 (27.0)	33 (26.8)	1.000
Vitamin K antagonist	13 (5.2)	9 (7.1)	4 (3.3)	0.273
Low molecular heparin	7 (2.8)	3 (2.4)	4 (3.3)	0.975

*Note:* Values are reported as mean ± standard deviation; or no. (%). Percentages may not total 100 because of rounding.

Abbreviations: 4‐IC: 4‐mm irrigated catheter; 8‐NIC: 8‐mm nonirrigated catheter; AF: Atrial fibrillation; CTI: Cavo‐tricuspid isthmus.

**TABLE 2:** Clinical characteristics of the propensity score‐matched population.

Characteristics after matching	All patients (*N* = 180)	4‐IC (*N* = 90)	8‐NIC (*N* = 90)	*p*
**Age**, year—mean ± SD	69.4 ± 9.4	69.2 ± 10.3	69.5 ± 8.4	0.815
**Sex**—no. (%)				
**Female**	45 (25.0)	22 (24.4)	23 (25.6)	1.000
**Cardiovascular risk factor**—no. (%)				
Arterial hypertension	128 (71.1)	65 (72.2)	63 (70.0)	0.860
Hypercholesterolemia	109 (60.6)	55 (61.1)	54 (60.0)	1.000
Diabetes mellitus	68 (37.8)	33 (36.7)	35 (38.9)	0.830
Tobacco	82 (45.6)	40 (44.4)	42 (46.7)	1.000
**Heart failure—**no. (%)				
Reduced or mid‐range ejection fraction	59 (32.8)	28 (31.1)	31 (34.4)	0.742
Preserved ejection fraction	28 (15.6)	13 (14.4)	15 (16.7)	0.814
**Coronary artery disease**	52 (28.9)	24 (26.7)	28 (31.1)	0.596
**AF history—**no. (%)	73 (40.6)	36 (40.0)	37 (41.1)	1.000
**Pacemaker—**no. (%)	28 (15.6)	14 (15.6)	14 (15.6)	1.000
**Redo CTI ablation—**no. (%)	10 (5.6)	4 (4.4)	6 (6.7)	0.752
**Medical history—**no. (%)				
Sleep apnea syndrome	27 (15.0)	14 (15.6)	13 (14.4)	1.000
Chronic obstructive pulmonary disease	48 (26.7)	23 (25.6)	25 (27.8)	0.864
Hypothyroidism–hyperthyroidism	27 (15.0)	13 (14.4)	14 (15.6)	1.000
Previous stroke history	15 (8.3)	7 (7.8)	8 (8.9)	1.000
Chronic kidney disease	52 (28.9)	25 (27.8)	27 (30.0)	0.864
Neoplasia history	23 (12.8)	11 (12.2)	12 (13.3)	1.000
**Echocardiography**				
Left ventricular ejection fraction, %—mean ± SD	52.49 ± 11.4	52.89 ± 11.6	52.09 ± 11.3	0.622
**Medical treatment**—no. (%)				
Antiarrhythmic class				
Class I	25 (13.9)	14 (15.6)	11 (12.2)	0.625
Class II	124 (68.9)	60 (66.7)	64 (71.1)	0.626
Class III—Sotalol	4 (2.2)	2 (2.2)	2 (2.2)	1.000
Class III—Amiodarone	43 (23.9)	22 (24.4)	21 (23.3)	1.000
Class IV	8 (4.4)	5 (5.6)	3 (3.3)	0.683
Antiplatelet	24 (13.3)	11 (12.2)	13 (14.4)	0.838
Anticoagulation	180 (100)	90 (100.0)	90 (100.0)	1.000
Apixaban	35 (19.4)	17 (18.9)	18 (20.0)	1.000
Dabigatran	20 (11.1)	9 (10.0)	11 (12.2)	0.814
Edoxaban	59 (32.8)	49 (38.9)	36 (29.3)	0.749
Rivaroxaban	50 (27.8)	24 (26.7)	26 (28.9)	0.880
Vitamin K antagonist	10 (5.6)	6 (6.7)	4 (4.4)	0.724
Low molecular heparin	6 (3.3)	3 (3.3)	3 (3.3)	1.000

*Note:* Values are reported as mean ± standard deviation; or no. (%). Percentages may not total 100 because of rounding.

Abbreviations: 4‐IC: 4‐mm irrigated catheter; 8‐NIC: 8‐mm nonirrigated catheter; AF: Atrial fibrillation; CTI: Cavo‐tricuspid isthmus.

### 3.2 Procedural Characteristics

Of the overall population, 125 underwent ablation with the 8‐mm nonirrigated tip catheter (8‐NIC group), and 128 with the 4‐mm irrigated‐tip catheter (4‐IC group), while 90 patients in both groups underwent ablation in the matched cohort. Electroanatomic mapping was used significantly more often in the 4‐IC group (*p* < 0.001). Successful bidirectional block was achieved in 97.8% of patients in both groups of propensity score‐matched patients (*p* = 1.000). One flutter recurrence within 24 h was observed in the 8‐NIC group, while none occurred in the 4‐IC group (*p* = 1.000). No major procedure‐related serious adverse event was reported. Procedural characteristics of the propensity score‐matched population are detailed in Table 3.

**TABLE 3:** Procedural characteristics of the propensity score‐matched population.

Characteristics	All patients (*N* = 180)	4‐IC (*N* = 90)	8‐NIC (*N* = 90)	*p*
Acute success bidirectional block—no. (%)	176 (97.8)	88 (97.8)	88 (97.8)	1.000
Electroanatomic mapping—no. (%)	86 (47.8)	71 (78.9)	15 (16.7)	**< 0.001**
Duration of radiofrequency application, sec—mean ± SD	320 ± 162	322 ± 156	319 ± 166	0.897
Early follow‐up (24 h)				
Flutter recurrence—no. (%)	1 (0.5)	0 (0)	1 (1.1)	1.000
Procedure‐related serious adverse events—no. (%)	0 (0.0)	0 (0.0)	0 (0)	1.000

*Note:* Values are reported as mean ± standard deviation; or no. (%). Percentages may not total 100 because of rounding.

Abbreviations: 4‐IC: 4‐mm irrigated catheter; 8‐NIC: 8‐mm nonirrigated catheter.

### 3.3 Long‐term Follow‐up

**FIGURE 1:**
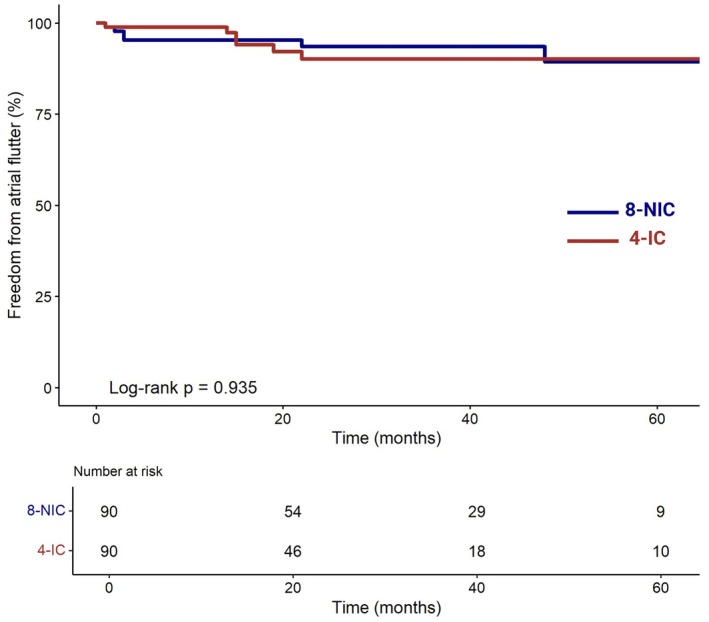
Kaplan–Meier analysis. Kaplan–Meier analysis demonstrating long‐term freedom from typical atrial flutter. 4‐IC: 4‐mm irrigated catheter; 8‐NIC: 8‐mm nonirrigated catheter; AF: Atrial fibrillation.

The mean follow‐up duration for the entire cohort was 27.0 ± 20.1 months (26.7 ± 19.6 months in the 4‐IC group vs. 28.7 ± 20.6 months in the 8‐NIC group, *p* = 0.529). During follow‐up, 93.3% (*n* = 84) of patients in the 4‐IC group remained free from typical atrial flutter, compared to 93.3% (*n* = 84) in the 8‐NIC group (log rank *p* value = 0.935, Figure 1). Atrial fibrillation occurred in 23.9% (*n* = 43) of patients without prior AF history, with no significant difference between groups (*p* = 1.000). Mortality and pacemaker implantation rates were comparable between the two groups (Table 4).

**TABLE 4:** Long‐term follow‐up of the propensity score‐matched population.

Characteristics	All patients (*N* = 180)	4‐IC (*N* = 90)	8‐NIC (*N* = 90)	*p*
Follow‐up duration, months—mean ± SD	27.7 ± 20.1	26.7 ± 19.6	28.7 ± 20.6	0.529
Freedom from atrial flutter recurrence—no. (%)	168 (93.3)	84 (93.3)	84 (93.3)	1.000
Newly diagnosed AF—no. (%)	43 (23.9)	22 (24.4)	21 (23.3)	1.000
Pacemaker implantation in the years following ablation—no. (%)	31 (17.2)	14 (15.6)	17 (18.9)	0.689
Death—no. (%)	24 (13.3)	12 (13.3)	12 (13.3)	1.000
Cardiovascular cause—no. (%)	4 (2.2)	2 (2.2)	2 (2.2)	1.000

*Note:* Values are reported as mean ± standard deviation; or no. (%). Percentages may not total 100 because of rounding.

Abbreviations: 4‐IC: 4‐mm irrigated catheter; 8‐NIC: 8‐mm nonirrigated catheter; AF: Atrial fibrillation.

## 4 Discussion

Our findings demonstrate that both the 8‐mm gold‐tip nonirrigated catheter and the 4‐mm irrigated‐tip catheter, applied in a high‐power, standard‐duration setting (60 W for a minimum of 30 s and 45 W for a minimum of 30 s, respectively), achieve similar rates of acute success, safety, and long‐term freedom from typical atrial flutter.

CTI ablation remains the treatment of choice for typical atrial flutter, with prior studies showing that large‐tip or irrigated‐tip catheters produce more effective lesions than standard 4‐mm catheters, while maintaining safety (Jais et al. 2000; Tsai et al. 1999). This improved efficacy is attributed to deeper and more contiguous lesions along the CTI, facilitating durable bidirectional block.

Comparisons of 8‐mm and irrigated‐tip catheters in previous studies have yielded conflicting results mainly due to variations in study designs, catheter types (including externally and internally irrigated catheter), and ablation mode settings; but it appears to perform equally regarding success rate and safety (Da Costa et al. 2005; Ilg et al. 2011; Iori et al. 2014; Sacher et al. 2007; Schreieck et al. 2002). Regarding the 8‐mm gold‐tip catheter used in this study, Sacher et al. reported comparable results to a 4‐mm externally irrigated catheter (ThermoCool, Biosense) in terms of effectiveness, radiofrequency, and fluoroscopy times (Sacher et al. 2007). Similarly, De Ruvo et al. demonstrated that the 8‐mm gold‐tip catheter performed equivalently to a 3.5‐mm open‐irrigated gold‐tip catheter (AlCath Flutter eXtra, Biotronik) (De Ruvo et al. 2018). Our study adds further evidence by showing that the 8‐mm gold‐tip catheter achieves outcomes comparable to the 4‐mm open‐irrigated‐tip catheter in a high‐power setting.

High‐power ablation strategies have recently gained attention, particularly in atrial fibrillation ablation protocols, for their ability to shorten lesion times while maintaining efficacy (Li et al. 2021). Usually in CTI ablation with a 4‐mm irrigated‐tip catheter, the power setting is typically limited to 30 W due to the risk of steam pops at higher levels. High power at 50 W for 9–20 s (Golian et al. 2020; Kwon et al. 2020; Tscholl et al. 2020) and very‐high power at 90 W for 4 s (Schillaci et al. 2022) have demonstrated results comparable to conventional methods. In our study, a setting of 45 W for a minimum of 30 s was applied without significant safety concerns. For 8‐mm nonirrigated catheters, conventional power settings are generally around 50 W, although manufacturer recommendations vary. Specifically for gold‐tip catheters, no formal manufacturer recommendation exists. In the AURUM 8 trial, a median power of 52 W was delivered in temperature‐controlled mode (Lewalter et al. 2011), while Sacher et al. used power limited to 50 W under similar conditions (Sacher et al. 2007). Our protocol extended this to 60 W for a minimum of 30 s without major complications, demonstrating its safety. Our high‐power, standard‐duration ablation protocol proved both safe and effective, achieving > 90% acute bidirectional block rates in line with existing data on CTI ablation efficacy.

Long‐term outcomes further support these findings. With a median follow‐up of more than two years, both catheter groups demonstrated high freedom from recurrent typical atrial flutter. There were no significant differences in de novo atrial fibrillation, pacemaker implantation rates, or cardiovascular mortality, underscoring the durability of both strategies. Long‐term follow‐up also revealed that while recurrence of typical flutter is rare, new‐onset atrial fibrillation remains more frequent, which is comparable to existing data (De Bortoli et al. 2017).

The more frequent use of 3D‐electroanatomical mapping in the 4‐IC group, due to catheter integration with the manufacturer's mapping system (EnSite, Abbott), did not significantly influence procedural success or safety outcomes. Despite this variation, the results suggest that mapping system choice had a minimal impact on overall outcomes.

This study has limitations. Its nonrandomized design, with catheter choice determined by operator preference, introduces potential selection bias. Additionally, the single‐center setting and sample size limit the generalizability of the findings. Procedural and radiation times were not consistently collected; however, the higher use of electroanatomical mapping in the 4‐IC group could confound direct comparisons of these metrics.

## 5 Conclusion

In this single‐center prospective study, both the 8‐mm gold‐tip nonirrigated catheter and the 4‐mm irrigated catheter demonstrated equivalent performance in high‐power CTI ablation, achieving high acute success rates with minimal complications and comparable long‐term outcomes.

## Author Contributions

W.Z. and A.S. conceived the idea and design of the study. L.M. and A.S. were the operators. W.Z. collected the data and wrote the manuscript. L.M. and K.‐R.E. contributed to critical feedback. A.S. and K.‐R.E. supervised the study.

## Conflicts of Interest

The authors declare no conflicts of interest.

## Data Availability

The data that support the findings of this study are available from the corresponding author upon reasonable request.
